# Characterisation of microbial metabolite profiles across the gastrointestinal tract of horses fed on pasture, hay or oat-supplemented rations

**DOI:** 10.1371/journal.pone.0359277

**Published:** 2026-09-28

**Authors:** Milena Marie Santo, Martin Bachmann, Gerald Fritz Schusser, Monika Wensch-Dorendorf, Caroline Pisch, Mandy Bochnia, Liane Hüther, Karin Heinichen, Annette Zeyner

**Affiliations:** 1 Institute of Agricultural and Nutritional Sciences, Martin Luther University Halle-Wittenberg, Halle (Saale), Germany; 2 Faculty of Veterinary Medicine, University of Leipzig, Leipzig, Germany; 3 Friedrich Loeffler Institute, Federal Research Institute for Animal Health, Braunschweig, Germany; Ankara University: Ankara Universitesi, TÜRKIYE

## Abstract

Dietary composition substantially influences the intensity and site of microbial metabolite production in the gastrointestinal tract. These metabolites have numerous beneficial effects on equine health, but may also be involved in the development of gastrointestinal disorders. It remains unclear whether different feeding strategies, including starch supplementation, can alter microbial metabolite profiles along the gastrointestinal tract. This study aimed to compare the metabolite profiles of the microbial communities in the gastrointestinal tract of horses fed on pasture 24 h/day (PST; n = 4), hay *ad libitum* (HAY; n = 8) or hay *ad libitum* and oats at 1 g starch/kg body weight (BW)/meal (OS1; n = 6) or 2 g starch/kg BW/meal (OS2; n = 6). After the feeding period, the horses were euthanised and dissected. Digesta samples were taken from different sections of the gastrointestinal tract and analysed for dry matter content, pH, short chain fatty acids (SCFA), ammonia and lactate. Significant differences were found among the sections for each parameter (*P* < 0.001) demonstrating a clear separation between foregut and hindgut and between proximal and distal sections of the hindgut. The ration type significantly affected dry matter (*P* < 0.001), total SCFA (*P* = 0.011), n-butyrate (*P* = 0.015), n-valerate (*P* = 0.045) and iso-SCFA concentrations (*P* = 0.017), while ration × section interactions were also detected for dry matter (*P* = 0.001), n-butyrate (*P* = 0.012), n-valerate (*P* = 0.001), iso-SCFA (*P* < 0.001) and ammonia (*P* < 0.001). The PST group was characterised by higher total SCFA concentrations in digesta, indicating enhanced fermentative activity. Particularly in the distal hindgut sections, higher concentrations of n-valerate, iso-SCFA and ammonia suggested increased amino acid fermentation and a potentially altered microbial protein metabolism. The supplementation of oats affected microbial metabolites predominantly in the stomach and suggested higher proteolytic activities.

## Introduction

Horses have evolved to continuously consume fibre-rich, low energy feed. Microbial fermentation in the gastrointestinal tract (GIT) plays a key role in the utilisation of nutrients. In addition to the composition and activity of microbial communities, microbial metabolites, particularly short chain fatty acids (SCFA), lactate and ammonia, are crucial as they serve as energy sources and modulate intestinal physiology, local environmental conditions and mucosal integrity in the GIT [1–3]. Keeping horses on pasture, as probably the most natural way of feeding and husbandry, may support a balanced microbial community and a metabolite profile beneficial for gastrointestinal health. The limited research conducted indicated that horses grazing on pasture exhibit higher concentrations of total SCFA in faeces compared to stabled horses fed exclusively hay, suggesting a potential benefit from increased microbial fibre fermentation [4]. The positive effects of stimulated fermentation include improved nutrient utilisation, epithelial barrier function and pathogen inhibition, specifically in the hindgut [5]. However, the specific effects of feeding horses on pasture on metabolite profiles in individual sections of the digestive tract, particularly in the stomach and small intestines, have not yet been described.

In horse husbandry, access to pasture and forage feeding is often restricted. In contrast, nonstructural carbohydrates, such as starch, often exceed recommended proportions. This may promote various intestinal and systemic diseases [6,7]. Among the gastrointestinal sites potentially affected, the equine stomach deserves particular attention, as its anatomical and physiological characteristics make it highly vulnerable to feed-related alterations. The Equine Gastric Ulcer Syndrome (EGUS) is a highly prevalent multifactorial disease [8], with insufficient roughage intake and excessive consumption of cereal grains and other concentrates high in starch representing major risk factors [7,9]. High SCFA concentrations have been shown to impair gastric mucosal integrity [10]. In addition, horses fed more than 1 g starch/kg body weight (BW)/meal as oats showed a significantly increased risk of EGUS in a field study by Luthersson et al. [7], forming the basis of the current recommendations of the Society of Nutrition Physiology [11].

Oat starch is primarily digested in the small intestine, but evidence exists that pre-digestion occurs in the equine stomach through acid hydrolysis and microbial fermentation [12,13]. The remaining starch reaches the small intestine, where it is enzymatically digested and absorbed as glucose. Starch, which passes undigested through the small intestine, enters the large intestine, where it is fermented into SCFA by the resident microbiota. The resulting SCFA profile depends on the cereal type and ration composition [14–16]. A ration rich in fibre results in high levels of acetate (and butyrate), while an increased proportion of cereal grains in the ration shifts the SCFA pattern in favour of propionate, iso-valerate and n-valerate [14,17,18]. The latter results in a lower ratio of acetate and n-butyrate to propionate [19]. Although the precaecal digestibility of starch from oats is nearly complete [20], Harlow et al. [21] demonstrated significant shifts in the faecal bacterial microbiota even when starch was provided within current recommendations. Whether these microbial shifts are accompanied by changes in microbial metabolites along the GIT remains unknown. However, changes in microbial composition and microbial metabolites do not necessarily indicate gastrointestinal disorders but may also represent an adaptive response to dietary or seasonal variation in substrate availability for microbial fermentation [22,23]. Taken together, ration type is expected to influence both the intensity and the site of the production of microbial metabolites and their associated effects on gastrointestinal health.

This study aimed to characterise the metabolic response of the microbial communities in the GIT of horses when they are kept on pasture or were fed hay *ad libitum* with or without the supplementation of oats. The study assessed potential changes in microbial fermentation processes in the digestive tract associated with these rations through the analysis of the main fermentation products SCFA, lactate and ammonia. We hypothesised that supplementation of oats leads to alterations in microbial metabolites, particularly within the stomach, marked by an enhanced proportion of propionate and valerate.

## Materials and methods

### Ethical approval

The horses involved in this study were housed and managed at the Oberholz Farm of Teaching and Research, University of Leipzig, Großpösna, Germany. The experimental protocol received approval from the Saxon Federal Administration Authority (reg. no. TVV 63/21) and the use of animals in this study was reported in compliance with the ARRIVE guidelines.

### Animals and management

24 horses were included in this study, consisting of 11 females and 13 males with an age of 11 ± 6.0 years and an initial BW of 471 ± 63.4 kg prior to the experimental trial. The body condition score (BCS; 1–9 scale) [24] and cresty neck score (CNS; 0–5 scale) [25] were 5.1 ± 0.67 and 1.0 ± 0.70, respectively. No major changes in body condition were observed during the experiment. After examination by a veterinarian, all horses were considered free from lameness, acute infections, metabolic diseases and able to enter the trial, based on the external signs observed. If necessary, dental treatment was performed. Additionally, the horses underwent a gastroscopic examination of the stomach prior to the study. Pregnancy was ruled out in mares by ultrasound examination. All of the horses were dewormed with ivermectin in accordance with the manufacturer's instructions. The animals were continuously monitored by a veterinarian throughout the entirety of the trial.

### Rations

The horses were allocated to one of four feeding groups (Table 1). The following rations were applied: free access to pasture (PST; n = 4), hay *ad libitum* (HAY; n = 8), hay *ad libitum* and oats at 1 g starch/kg BW/meal (OS1; n = 6) and hay *ad libitum* and oats at 2 g starch/kg BW/meal (OS2; n = 6). Meadow hay was offered in large plastic containers. In a subset of six horses, individual hay intake in the stable was measured over five consecutive days and averaged 98.6 ± 10.7 g DM/kg metabolic BW. Horses from OS1 and OS2 were given whole oat grains of the cultivar ‘Apollon’ (Nordsaat Saatzucht GmbH, Schaprode, Germany), which is a yellow hulled oat variety, once a day in the morning. The oat grains contained 437 g starch/kg dry matter (DM). The starch dosage in OS1 was based on the recommendations of the Society of Nutrition Physiology [11] and doubled for OS2. The horses received their last portion of oats one hour prior to sedation, which was eaten in its entirety. The same batches of hay and oats were used throughout the entire study to reduce variation in feed composition between periods. All horses had *ad libitum* access to tap water and a salt lick. Horses of the HAY, OS1 and OS2 groups were housed in individual boxes (at least 16 m² each) bedded with straw and had access to a paddock in groups for several hours a day, where hay was available in hay nets. Horses of the PST group were kept together on pasture for 24 h/day after a period of adaptation. Adaptation to the pasture occurred by hourly grazing whereby the horses also received meadow hay, first during the hours in the stable and later also on pasture. During this time, the pasture area remained limited and was opened up to two hectares after complete conversion to pasture grass and in adaptation to the course of vegetation. After the adaptation period, no additional hay was offered to the horses of the PST group. The botanical composition corresponded to a typical Central German vegetation on managed permanent grassland. The botanical composition of the hay was not determined. However, as the hay originated from fields located in close proximity to the pasture, a comparable botanical composition can be expected. Details can be found in Bachmann et al. [26] and Table 2.

**Table 1:** Number of horses allocated to each ration and period.

	Period 1	Period 2	Period 3	Period 4
PST				4
HAY	2	2	2	2
OS1	2	1	2	1
OS2	2	2	1	1

Rations: PST, pasture 24 h/day (n = 4); HAY, hay *ad libitum* (n = 8); OS1, hay *ad libitum* and oats at 1 g starch/kg BW/meal (n = 6); OS2, hay *ad libitum* and oats at 2 g starch/kg BW/meal (n = 6). Abbreviations: BW, body weight.

**Table 2:** Chemical composition of experimental feed, summarised from Bachmann et al. [26].

Item	Matured Pasture	Hay	Oats
Dry matter	507	944	926
Crude protein	76	81	114
Precaecal digestible crude protein^1^	39	49	92
Crude fibre	316	366	102
Neutral detergent fibre	648	661	291
Acid detergent fibre	387	396	135
Nonstructural carbohydrates^2^	177	190	528^3^
Metabolizable energy^4^	6.9	6.2	12.7

Dry matter (DM) is given in g/kg, metabolized energy is given in MJ/kg DM and all other analytes in g/kg DM. For hay and oats, values are presented as means of the four experimental periods. Neutral and acid detergent fibre are expressed exclusive of residual ash. Neutral detergent fibre was treated with amylase.

^1^Calculated according to Zeyner et al. [27].

^2^Calculated as follows: non-structural carbohydrates = dry matter – crude ash – crude protein – crude lipids – neutral detergent fibre.

^3^Oats contained 437 g starch/kg DM.

^4^Calculated according to Kienzle and Zeyner [28] using dynamic estimates of renal energy loss per gram crude protein from Kuchler et al. [29].

### Experiment

The experiment was divided into four consecutive periods due to capacity restrictions. Individual feeding periods lasted between 34 and 73 days to allow the horses to adapt to the conditions, including a prolonged adaptation period for the horses transitioned from a hay-based ration to pasture. Horses remained assigned to the same diet throughout the study. All diets were represented in each period, with the exception of PST. The PST treatment was restricted to a single experimental period to ensure that all pasture-fed horses were exposed to a comparable vegetation stage and pasture conditions. To provide a reference group for the estimation of potential period effects, the HAY group was included with the same number of horses in each period (Table 1). Euthanasia, dissection and sampling were performed at the end of each period. Light sedation was initiated with detomidine and butorphanol (each 0.01 mg/kg BW intravenous (i.v.)) and deep sedation with a mixture of detomidine (0.04 mg /kg BW i.v.) and butorphanol (0.025 mg /kg BW i.v.). Anaesthesia was induced with diazepam (0.05 mg/kg BW i.v.) and ketamine (2.2 mg/kg BW i.v.) before the horses were euthanised with an overdose of pentobarbital (90 mg/kg BW i.v.). The horses were positioned on a dissection table and the abdominal cavity was opened along the linea alba. The digestive tracts were detached at the rectum, subsequently dissected from caudal to cranial along the mesentery and then cut at the duodenum situated in close proximity to the pylorus. The stomach was removed completely and opened along the greater curvature. The entire digesta from the pars nonglandularis (PNG) and pars glandularis (PG) were collected separately. In the next step, the small intestine was separated from the large intestine at the ileocaecal junction. Further digesta samples were collected from the following sections: centred jejunum (JEJ), caecum (CAE), colon ventrale dextrum (CVD), colon ventrale sinistrum (CVS), pelvic flexure (PFL), colon dorsale sinistrum (CDS), colon dorsale dextrum (CDD) and colon transversum (CTR). For these intestinal sections, digesta were collected in high quantities, depending on the available content of each section. Faecal samples were collected manually from the rectum (REC) post mortem. All digesta samples were taken under comparable conditions and within one hour after euthanasia. Digesta samples were immediately stored on ice and subsequently frozen at −20 °C until analysis.

### Biochemical analyses

For the determination of DM content, the freeze-dried samples were further dried at 105 °C for 3 hours. The digesta samples were diluted in a 1:1 ratio with deionised water as described by Zeyner et al. [30]. Then, the pH value was measured after 1h of incubation using a Mettler Toledo Seven Excellence unit with an InLab® Expert Pro electrode (Mettler Toledo GmbH, Greifensee, Switzerland). The digesta eluates were stored at −20 °C until further measurements were carried out. The concentration of ammonia was determined from the eluates using the method of Conway and Byrne [31]. For the analysis of SCFA, a Shimadzu GC2010 unit (Shimadzu Corp., Kyoto, Japan) fitted with a flame ionization detector was used. The eluates were centrifuged for 5 min at 4000 rpm, before internal standard (1.5 g 4-methyl-valeric acid/100 mL formic acid, 80%) was admitted to the supernatant. After a second cycle in the centrifuge (5 min at 4000 rpm), the supernatant of the samples was filled in 2-mL vials and injected on-column. The gas chromatographic operation details were as follows: SGE BP21 column (30 m × 0.53 mm × 0.5 µm) (Trajan Scientific and Medical, Ringwood, Australia) with helium acting as carrier and make-up gas; injection temperature and volume set to 180 °C and 0.5 µL, respectively; constant pressure mode at 22.7 kPa (i.e., 29.7 cm/s linear velocity and 3.64 mL/min column flow); initial oven temperature set to 85 °C, raised up to 200 °C by 8 °C/min to be held for 6 min; detection temperature of 200 °C. An external 6-point standard calibration was carried out to calculate the target SCFA concentrations. The branched-chain fatty acids iso-butyrate and iso-valerate are collectively referred to as iso-SCFA due to their similar pattern and low concentrations. The ratio of acetate (C2) and n-butyrate (C4) to propionate (C3) was calculated and described as (C2 + C4)/C3. The concentration of lactate was measured on a liquid chromatography device fitted with a photodiode array detector (Shimadzu Corp., Kyoto, Japan), 300 mm × 7.8 mm Rezex ROA-Organic Acid H+ separation column and Security Guard cartridge, Carbo-H 4 × 3 mm (Phenomenex Ltd., Aschaffenburg, Germany). Digesta-innate water was mixed with 0.016 N sulphuric acid in a ratio of 1:1 (0.8 mL each) and stored at −20 °C. Samples were mixed with a benchtop vortexer and centrifuged for 10 min at 14000 rpm at 20 °C. The supernatant was diluted with 0.016 N sulphuric acid in a 1:2 ratio (2 mL in total). The samples were filtered through a syringe filter and injected onto the chromatograph with a volume of 20 μL. Initial oven temperature was set to 45 °C. The 0.016 N sulphuric acid served as eluent at 0.6 mL/min isocratic flow. Lactate was detected at 210 nm. 1 g L-(+)-lactic acid in 200 mL 0.016 N sulfuric acid served as standard stock solution. An 8-point external calibration was carried out in a range of 0.2 to 4 g/L. To relate the measured concentrations of SCFA, lactate and ammonia to the digesta-innate water, the equation proposed by Zeyner et al. [30] was used taking the DM content of each sample into account.

### Statistical analysis

Statistical analysis was performed using SAS 9.4 (SAS Institute Inc., Cary, NY, USA). Data were analysed using the MIXED procedure according to the following linear mixed model: *y*_*ijkl*_ = *μ* + *α*_*i*_ + *β*_*j*_ + *γ*_*k*_ + *βγ*_*jk*_ + *a*_*l*_ + *ε*_*ijkl*_, where *y*_*ijkl*_ is the dependent variable; *μ* is the overall mean; *α*_*i*_ is the fixed effect of period *i* (*i* = 1, …, 4); *β*_*j*_ is the fixed effect of ration *j* (*j* = 1, …, 4, where 1 = PST, 2 = HAY, 3 = OS1, 4 = OS2); *γ*_*k*_ is the fixed effect of gastrointestinal section *k* (*k* = 1, …, 11, where 1 = pars nonglandularis, 2 = pars glandularis, 3 = jejunum, 4 = caecum, 5 = colon ventrale dextrum, 6 = colon ventrale sinistrum, 7 = pelvic flexure, 8 = colon dorsale sinistrum, 9 = colon dorsale dextrum, 10 = colon transversum, 11 = rectum); *βγ*_*jk*_ is the interaction of fixed effects *β*_*j*_ and *γ*_*k*_; *a*_*l*_ is the random effect of animal *l* (*l* = 1, …, 23) to account for repeated measurements within animal; and *ε*_*ijkl*_ is the random residual effect with *ε*_*ijkl*_ ~ N(0,σ^2^*ε,*_*k*_) which means that the heterogeneous residual variances were allowed for the different intestinal sections, resulting in a heterogeneous compound symmetry covariance structure. For pairwise comparisons of the main effects, Tukey-Kramer adjustment was applied. No multiple comparison adjustment was applied to pairwise comparisons of the ration × section interaction. The studentised residuals were confirmed to be normally distributed using the procedure UNIVARIATE, specifically the Kolmogorov-Smirnov test and visual examination of the histograms. The concentrations of individual SCFA (except acetate), lactate and ammonia were subjected to logarithmic transformation in order to ensure normal distribution. Differences between least squares means with *P* < 0.05 were considered significant. One horse (no. 24) of the feeding group OS2 was excluded from statistical analysis, because this horse consumed its morning oat meal throughout the entire day, and this did not correspond to the experimental setup. The results are presented as arithmetic means with standard deviation.

## Results

The raw data are provided in S1 Table. The results of the tests of fixed effects are summarised in Table 3. Significant ration × section interactions were observed for DM (*P* = 0.001), n-butyrate (*P* = 0.012), n-valerate (*P* = 0.001), iso-SCFA (*P* < 0.001) and ammonia (*P* < 0.001), as further demonstrated in Fig 1. The effect of ration was found to be significant for the parameters DM, total SCFA, n-butyrate, n-valerate and iso-SCFA and revealed a tendency for lactate (*P* = 0.064) and ammonia (*P* = 0.051). Highly significant differences were observed among the various anatomical sections for all examined parameters, resulting in a characteristic pattern along the GIT (*P* < 0.001; Table 3). The course of the different parameters along the GIT is summarized in Table 4. A significant effect of the period was observed on DM (*P* < 0.001), pH value (*P* = 0.045), propionate (*P* = 0.006), n-butyrate (*P* = 0.008) and n-valerate (*P* = 0.028).

**Table 3:** *P*-values of tests of fixed effects displaying the effect of ration and gastrointestinal section on dry matter content, pH value, and the concentrations of, SCFA, lactate, ammonia and the (C2 + C4)/C3 ratio.

	Ration	Section	Ration*Section
Dry matter	**< 0.001**	**< 0.001**	**0.001**
pH	0.846	**< 0.001**	0.125
SCFA	**0.011**	**< 0.001**	0.166
Acetate	0.226	**< 0.001**	0.507
Propionate	0.075	**< 0.001**	0.197
n-Butyrate	**0.015**	**< 0.001**	**0.012**
n-Valerate	**0.045**	**< 0.001**	**0.001**
iso-SCFA	**0.017**	**< 0.001**	**< 0.001**
Lactate	0.064	**< 0.001**	0.466
Ammonia	0.051	**< 0.001**	**< 0.001**
(C2 + C4)/C3	0.487	**< 0.001**	0.444

(C2 + C4)/C3, ratio of acetate and n-butyrate to propionate; iso-SCFA, sum of iso-butyrate and iso-valerate. Abbreviations: SCFA, short chain fatty acids.

**Table 4:** Dry matter content (g/kg), pH value and concentration of SCFA, lactate, ammonia (mmol/L) and the (C2 + C4)/C3 ratio along the gastrointestinal tract of horses.

	PNG	PG	JEJ	CAE	CVD	CVS	PFL	CDS	CDD	CTR	REC	s.d.
**DM**	222^a^	170^bc^	66^f^	84^e^	114^d^	115^d^	104^d^	109^d^	110^d^	152^c^	182^b^	1.86 - 9.65
**pH**	5.3^f^	3.0^e^	7.7^a^	7.2^abc^	6.6^d^	6.9^bcd^	7.2^ab^	7.1^bc^	6.9^cd^	6.8^cd^	6.8^bcd^	0.059 - 0.20
**SCFA**	19.3^e^	9.4^f^	3.8^g^	84.4^b^	114.8^a^	109.6^a^	91.3^ab^	93.1^ab^	93.5^ab^	61.5^c^	44.4^d^	0.7 - 6.97
**Acetate**	17.6^e^	8.4^f^	3.6^g^	59.4^b^	79.6^a^	75.4^ab^	64.5^ab^	71.1^ab^	70.3^ab^	45.3^c^	33.3^d^	0.80 - 4.68
**Propionate**	0.7^f^	0.2^f^	n.d.	17.0^bc^	21.3^a^	20.7^ab^	16.0^bc^	14.3^c^	14.3^c^	9.8^d^	7.1^e^	0.2 - 5.46
**n-Butyrate**	0.5^e^	0.4^e^	0.2^e^	6.3^b^	10.8^a^	10.0^a^	6.2^b^	5.7^b^	5.7^b^	3.2^c^	2.1^d^	0.02 - 0.45
**n-Valerate**	0.02^d^	<0.01^d^	n.d.	0.32^bc^	0.65^a^	0.76^a^	0.67^a^	0.79^a^	0.75^a^	0.40^b^	0.25^c^	0.01 - 0.45
**iso-** **SCFA**	0.13^f^	0.10^f^	n.d.	0.61^e^	2.22^bc^	2.69^abc^	2.80^ab^	3.55^a^	3.55^a^	1.89^c^	1.24^d^	0.14 - 1.60
**Lactate**	57.1^a^	21.3^b^	3.9^c^	n.d.	n.d.	n.d.	n.d.	n.d.	n.d.	n.d.	n.d.	4.1 - 34.2
**Ammonia**	8.2^cd^	4.6^e^	5.9^de^	3.5^f^	13.9^abcd^	14.1^ab^	13.7^bc^	21.3^a^	19.4^a^	11.4^b^	10.8^bc^	1.3 - 13.1
(**C2** **+** **C4)/C3**	74.8^a^	72.5^a^	n.d.	3.8^d^	4.3^cd^	4.3^cd^	4.5^cd^	5.5^b^	5.5^b^	5.0^bc^	5.2^bc^	0.41 - 97.2

Sections: PNG, gastric pars nonglandularis; PG, gastric pars glandularis; JEJ, jejunum; CAE, caecum; CVD, colon ventrale dextrum; CVS, colon ventrale sinistrum; PFL, pelvic flexure; CDS, colon dorsale sinistrum; CDD, colon dorsale dextrum; CTR, colon transversum; REC, rectum. Abbreviations: DM, dry matter; iso-SCFA, iso-short chain fatty acids (the sum of iso-butyrate and iso-valerate); (C2 + C4)/C3, ratio of acetate and n-butyrate to propionate; n.d., not detectable; SCFA, short chain fatty acids; s.d., standard deviation. ^abc^Superscripts indicate differences among the sections within parameters with *P* < 0.05.

**Fig 1:**
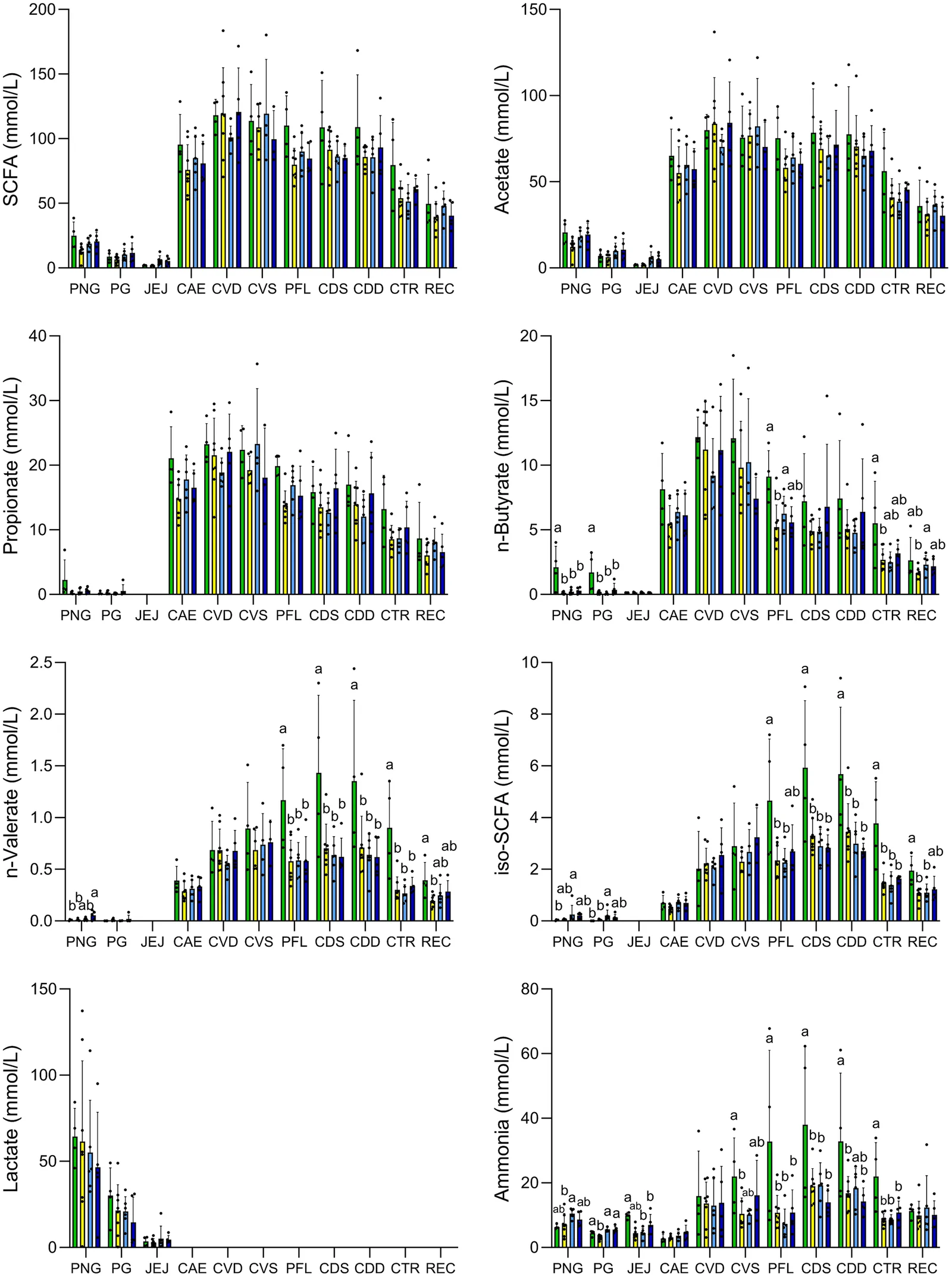
Concentration of organic acids and ammonia in the digesta collected from different sections of the gastrointestinal tract of horses receiving one of four rations. Values are presented as means. Error bars indicate the standard deviation. Rations: PST, pasture (24 h/d, n = 4, green); HAY, hay ad lib (n = 8, yellow); OS1, hay *ad libitum* and oats at 1g starch/kg BW/meal (n = 6, light blue); OS2, hay *ad libitum* and oats at 2 g starch/kg BW/meal (n = 5, dark blue). Sections: PNG, gastric pars nonglandularis; PG, gastric pars glandularis; JEJ, jejunum; CAE, caecum; CVD, colon ventrale dextrum; CVS, colon ventrale sinistrum; PFL, pelvic flexure; CDS, colon dorsale sinistrum; CDD, colon dorsale dextrum; CTR, colon transversum; REC, rectum. Abbreviations: BW, body weight; iso-SCFA, sum of iso-butyrate and iso-valerate; SCFA, short chain fatty acids. ^abc^Superscripts indicate with *P* < 0.05 different means within the same gastrointestinal section among all rations.

The content of DM and the pH value are displayed in Table 5. The highest DM content was found in PNG (*P* < 0.05) followed by a rapid decrease and lowest DM in JEJ (*P* < 0.01). Along the hindgut, DM increased up to the rectum with an average DM content of 182 g/kg. In general, DM contents were higher in horses that consumed oats (*P* ≤ 0.001). The DM content exhibited a significant ration × section interaction (*P* = 0.001, Table 5).

**Table 5:** Dry matter content (g/kg) and pH values in digesta along the gastrointestinal tract of horses allocated to different rations.

	Section	PST	HAY	OS1	OS2
Dry matter	PNG	**162**^**b**^ (16.6)	**158**^**b**^ (53.8)	**276**^**a**^ (25.6)	**287**^**a**^ (70.4)
	PG	**136**^**b**^ (11.5)	**134**^**b**^ (37.9)	**192**^**ab**^ (10.0)	**211**^**a**^ (39.6)
	JEJ	**51** (4.8)	**66** (8.2)	**70** (12)	**73** (13)
	CAE	**75** (8.2)	**84** (7.6)	**92** (18)	**81** (16)
	CVD	**92**^**b**^ (16)	**115**^**ab**^ 13.4)	**120**^**a**^ (7.41)	**123**^**a**^ (21.7)
	CVS	**96** (14)	**121** (14.2)	**121** (11.7)	**127** (16.9)
	PFL	**102**^**ab**^ (6.84)	**95**^**b**^ (15)	**110**^**a**^ (11.8)	**104**^**ab**^ (14.8)
	CDS	**102**^**ab**^ (10.5)	**101**^**b**^ (11.2)	**115**^**a**^ (16.4)	**112**^**ab**^ (18.2)
	CDD	**110** (11.8)	**106** (8.80)	**107** (12.7)	**114** (10.6)
	CTR	**139**^**b**^ (12.9)	**146**^**b**^ (7.83)	**163**^**a**^ (9.09)	**156**^**a**^ (26.1)
	REC	**167**^**b**^ (11.7)	**174**^**b**^ (15.9)	**194**^**a**^ (16.2)	**189**^**a**^ (12.0)
pH	PNG	**4.4** (0.43)	**5.3** (1.1)	**6.0** (0.67)	**5.2** (0.61)
	PG	**2.3** (0.73)	**2.7** (0.74)	**3.7** (0.70)	**3.2** (1.3)
	JEJ	**7.6** (0.23)	**7.6** (0.44)	**7.6** (0.46)	**7.5** (0.57)
	CAE	**6.6** (0.59)	**7.3** (0.50)	**7.4** (0.38)	**7.3** (0.55)
	CVD	**6.4** (0.083)	**6.5** (0.66)	**6.7** (0.66)	**6.8** (0.78)
	CVS	**6.5** (0.50)	**6.8** (0.52)	**6.8** (0.19)	**7.0** (0.23)
	PFL	**6.9** (0.58)	**7.3** (0.29)	**7.2** (0.19)	**7.3** (0.36)
	CDS	**6.8** (0.38)	**7.0** (0.35)	**7.2** (0.38)	**7.1** (0.23)
	CDD	**6.9** (0.47)	**6.9** (0.32)	**6.9** (0.25)	**7.0** (0.19)
	CTR	**6.8** (0.33)	**6.8** (0.56)	**6.8** (0.44)	**6.7** (0.20)
	REC	**6.9** (0.51)	**7.1** (0.99)	**6.5** (0.47)	**6.7** (0.14)

Rations: PST, pasture 24 h/day (n = 4); HAY, hay *ad libitum* (n = 8); OS1, hay *ad libitum* and oats at 1 g starch/kg BW/meal (n = 6); OS2, hay *ad libitum* and oats at 2 g starch/kg BW/meal (n = 5). Sections: PNG, gastric pars nonglandularis; PG, gastric pars glandularis; JEJ, jejunum; CAE, caecum; CVD, colon ventrale dextrum; CVS, colon ventrale sinistrum; PFL, pelvic flexure; CDS, colon dorsale sinistrum; CDD, colon dorsale dextrum; CTR, colon transversum; REC, rectum. ^abc^Superscripts indicate with *P* < 0.05 different means within a section among the rations. Standard deviation is given in brackets.

The pH value was lowest in PG, with an average of 3.0, while the highest value was found in JEJ at 7.7 (Table 4). Only minor differences were detected between the hindgut sections. The ration had no significant influence on the pH value in the digesta; only numerically in the gastric digesta. Especially in the PG, the pH appeared to be lower with roughage only rations (PST < HAY) than with oat supplementation.

Within the foregut, the concentration of total SCFA was highest in PNG (*P* < 0.001) and lowest in JEJ (*P* < 0.001). The overall highest concentrations were found in the hindgut between CAE and CDD (*P* < 0.01), with peaks in CVD and CVS. In the CAE, the total SCFA concentration was significantly lower than in the CVD (*P* = 0.001) and CVS (*P* = 0.033). A significant decrease was detected from CDD to REC (*P* < 0.05). Regarding the influence of the ration, the concentration of total SCFA was highest with PST, especially compared to HAY (*P* = 0.006) and OS1 (*P* = 0.045), and tended to be higher than OS2 (*P* = 0.060). The concentrations of the two most abundant SCFAs, acetate and propionate, exhibited the same trend along the GIT, peaking in CVD, followed by the CVS. The ration had no significant influence on acetate and propionate concentration in the digesta. Propionate was not detectable in the JEJ, as well as n-valerate and the iso-SCFA. Regarding n-butyrate, n-valerate and iso-SCFA concentration, the ration had significant effect. Overall, higher concentrations of these SCFA were detected in PST compared to HAY (*P* = 0.018, *P* = 0.045 and *P* = 0.014, respectively). For n-butyrate, a concentration peak was found in the ventral colon (CVD and CVS; *P* < 0.05). Besides a significant ration × section interaction (Table 3 and Fig 1), n-butyrate was higher in PST compared to HAY (*P* = 0.018) in most of the cases. Along the GIT, the n-valerate concentration peaked between CVD and CDD (*P* < 0.05). While in the PNG, the concentration of n-valerate was higher in OS2 than in HAY (*P* = 0.015) and PST (*P* = 0.010), the n-valerate concentration was by far highest in PST from the PFL to the CTR (*P* < 0.05). The branched-chain SCFA iso-butyrate and iso-valerate exhibited a similar pattern and were evaluated together as iso-SCFA. In contrast to the more abundant SCFA, the iso-SCFA clearly reached their maximum levels in the dorsal colon. In PNG and PG, the concentration the iso-SCFA was higher in OS1 than in PST (*P* = 0.018 and *P* < 0.001, respectively). Additionally, in the PG, the concentrations of the iso-SCFA were found to be significantly lower in HAY than in OS1 (*P* < 0.001). The opposite trend was observed in the lower colon sections caudal to the pelvic flexure (PFL to REC), where iso-SCFA in PST were clearly increased compared to the other rations (Fig 1). The molar percentages of SCFA given as means with standard deviation as well as the results of the F-test are summarised in S2 and S3 Table. Significant effects confirm the differences in SCFA concentrations, with particular regard to the molar percentages of n-butyrate in the stomach on PST (*P* < 0.05) showing an enhanced effect compared to the concentrations. The (C2 + C4)/C3 ratio was not affected by the ration or the ration × section interaction (Table 3). However, a clear difference was revealed among three major areas in the GIT: the ratio was 72.5 to 74.8 in the stomach, 3.8 to 4.3 in CAE and the ventral colon and 5.0 to 5.5 from the dorsal colon to the REC (*P* < 0.05, Table 4). The PFL was found intermediate with 4.5, separating the proximal and the distal large intestine.

A significant reduction in lactate concentration was observed from PNG to PG (*P* = 0.015) and further to the JEJ (*P* < 0.001). A statistical analysis was not conducted in the hindgut due to the fact that lactate was not detectable in 59.0% of samples (40.6% in PST, 74.2% in HAY, 58.7% in OS1, 50.0% in OS2) of the samples. The type of ration tended to have an influence on the lactate concentration (*P* = 0.064) with higher concentrations found in PST compared to OS2 (*P* = 0.060) and HAY (*P* = 0.058).

Similar to the pattern of the iso-SCFA, the concentration of ammonia was highest in the dorsal colon. The effect of ration was not statistically significant (*P* = 0.051). However, pairwise comparisons of the estimated least squares means revealed a significant lower concentration with HAY compared to PST (P = 0.033). The ration × section interaction was significant (*P* < 0.001). In the PNG, ammonia concentration was lower in HAY than in OS1 (*P* = 0.022). In the PG, ammonia concentration was consistently lowest in HAY (*P* < 0.05). In the JEJ, the CVD and the hindgut sections thereafter, ammonia concentration was highest in PST. This was significant from CVS to CTR (Fig 1; *P* < 0.05).

A principal component analysis (PCA) was performed based on the results of the individual SCFA, ammonia and the pH values to reflect shifts in the microbial fermentation profile. This analysis indicates that the stomach sections clearly cluster apart from the hindgut sections and that within the hindgut, the sections proximal and distal of the PFL cluster separately (Fig 2A). The feeding groups did not form distinct clusters (Fig 2B).

**Fig 2:**
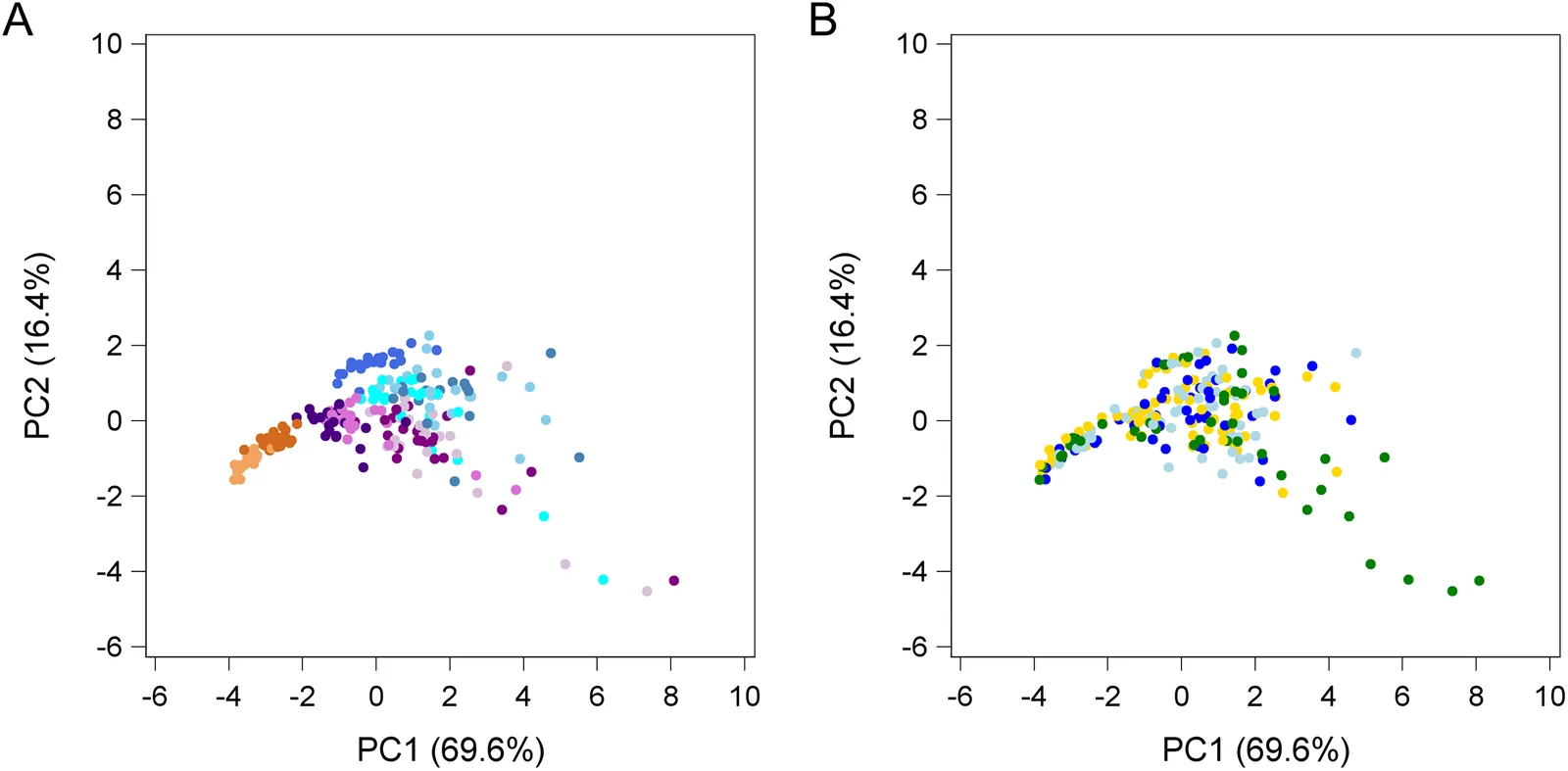
Principal component analysis (PCA) of microbial metabolite profiles in digesta samples collected from different sections of the gastrointestinal tract of horses receiving one of four rations. The PCA is based on the correlation of standardized data of pH value, individual SCFA (acetate, propionate, n-butyrate, n-valerate and iso-SCFA) and ammonia differentiated by section (A) or type of ration (B). Sections: PNG, gastric pars nonglandularis (dark orange); PG, gastric pars glandularis (light orange); CAE, caecum (royal blue); CVD, colon ventral dextrum (light blue); CVS, colon ventral sinistrum (steel blue); PFL, pelvic flexure (turquoise); CDS, colon dorsale sinistrum (light lavender); CDD, colon dorsale dextrum (purple); CTR, colon transversum (pink); REC, rectum (dark purple). Rations: PST, pasture (24 h/d, n = 4, green); HAY, hay *ad libitum* (n = 8, yellow); OS1, hay *ad* *libitum* and oats at 1g starch/kg BW/meal (n = 6, light blue); OS2, hay *ad* *libitum* and oats at 2 g starch/kg BW/meal (n = 5, dark blue). Abbreviations: BW, body weight; iso-SCFA, sum of iso-butyrate and iso-valerate; SCFA, short chain fatty acids.

## Discussion

The GIT of horses is evolutionarily adapted to continuous feed intake. Horses therefore practice ‘foraging’ with breaks of no more than 3–4 hours between feed intake [32]. Taylor et al. [33] observed Icelandic horses in a large field and found that they spend 12–17 hours per day foraging. Long feeding breaks and low fibre intake are therefore often associated with disorders such as EGUS, dysbiosis or altered behaviours like oral stereotypies [5,7,34]. Due to this, horses in this study had *ad libitum* access to hay, straw from the bedding or pasture to avoid the impact of insufficient fibre intake or stress caused by prolonged feeding breaks. As the individual forage intake was not quantified for all horses, inter-individual variation in hay and straw consumption cannot be excluded.

As demonstrated by Bachmann et al. [26], the analysis of the nutrient concentrations in the feed revealed typical results for Central Germany. For OS1 and OS2, the desired starch levels were successfully achieved. By the end of the feeding period, the pasture had matured, resulting in only minor differences between the hay and the pasture samples regarding the nutrient composition.

The DM content of the digesta within the equine GIT reflects the interaction between type of feed, water balance, passage rate and microbial fermentation, and is therefore considered as an important functional parameter to assess gastrointestinal health. In the present study, DM content was highest in the stomach and lowest in the small intestine. Moreover, it increased in the hindgut sections towards the rectum. This pattern of DM contents confirms results of earlier studies [35]. The higher DM contents, which were observed when oats were fed, are also in line with previous studies that demonstrated a positive association between concentrate intake and increased DM contents in digesta [18,21,36]. The impact on DM may be attributed to different factors, including reduced salivation when concentrates are consumed [36] and enhanced water-binding capacity of roughage compared to concentrates [37]. It is well documented that horses consume more water when forage-only rations are fed compared to rations including cereal grains [38–40]. Higher concentrations of SCFA may enhance sodium absorption and, consequently, intestinal water resorption [41]. However, other factors seem to have a greater influence on DM content. The relatively low DM content in the rectum ranging from 167 to 194 g/kg, especially in PST and HAY groups, compared to 180–200 g/kg [38] and 206–232 g/kg [30] in others studies might be explained by the *ad libitum* access to either pasture or hay. As Zeyner et al. [30] demonstrated, an increased hay intake is associated with a decrease in faecal DM content within a physiological range.

Many gastrointestinal disorders in horses are associated with changes in pH value, like the development of EGUS [9]. While the pH value in the equine stomach is predominantly dependent on hydrochloric acid production in the glandular region and to a lesser proportion on the microbial fermentation, the pH value in the hindgut is strongly influenced by microbial metabolites as these occur in much higher concentrations in these sections. The formed SCFA play a major role, with ammonia, for instance, counteracting the organic acids in case of buffering, resulting in a nearly pH-neutral environment in the intestine. Our results, demonstrating low pH in the stomach and high pH in the JEJ, are consistent with findings of other studies, in which pH ranged from 1.3 to 5.1 in the stomach, 5.9 to 7.8 in the small intestine and 5.8 to 6.9 in the hindgut [15,17,30,42]. Interestingly, pH was not significantly affected by the ration, even when horses received up to 2 g starch/kg BW/meal. This contrasts with previous reports of reduced intestinal pH associated with starch containing meals [16,43–45]. This may be due to the combination of oat starch’s high precaecal digestibility, limiting the starch flow to the hindgut, and *ad libitum* provision of forage. These factors likely contributed to maintaining a stable pH in the digesta. Nevertheless, numerically higher pH values found in gastric digesta of horses fed oats may be attributed to insufficient soaking of the digesta with the produced acid. This effect may result from the physicochemical properties of oats compared to roughage [37].

The primary metabolites of microbial fermentation in equines’ GIT are SCFA, which serve as a pivotal energy source. Horses are capable to cover 60–70% of their energy maintenance requirements through SCFA [46,47]. In addition, SCFA act as modulators for the immune system, provide energy for colonocytes and promote the intestinal barrier integrity [1–3,48]. The concentration of total SCFA in digesta varies considerably among different anatomical sections. This variation reflects differences in microbial density and composition, substrate availability, transit time as well as SCFA production and epithelial absorption. Nevertheless, it is accepted as a functional parameter for microbial fermentation activity. The SCFA concentration in the foregut is comparatively low due to rapid digesta transit and a less diverse microbial community [49–51]. However, especially in the PNG initial fermentation is reflected by the concentrations of SCFA, lactate and ammonia [15,52]. In contrast, the hindgut provides different conditions with longer retention time, different substrates for fermentation and a higher pH compared to the stomach. This leads to distinct differences in the composition and the activity of the microbiota among the gastrointestinal sections [49,53]. Regardless of the foregut, it is documented that different hindgut sections also vary in terms of their microbial composition and activity [54,55]. It is assumed that the primary site of fermentation and, consequently, fibre degradation is in the right ventral colon [15,56,57]. Our results confirm this, revealing the highest concentration of total SCFA in CVD with 114.8 mmol/L. An increase in total SCFA concentration is often associated with concentrate intake [15,44]. Our findings indicate highest total SCFA concentration in the digesta of horses on PST. On the other hand, total SCFA concentration in the digesta was lowest in the HAY group. This reflects a higher microbial fermentative activity on pasture, probably resulting in an enhanced energy supply. These observations could be attributed either to a higher amount of readily fermentable carbohydrates in the upper GIT of the PST group and/or to more efficient fibre degradation along their whole GIT. In this experiment, Bachmann et al. [26] found evidence for an initial degradation of fructans and plant cell wall constituents in the stomach, particularly when horses were kept on pasture. Notably, fructan degradation in the stomach did not occur in the OS2 group. An initial fibre degradation in the precaecal sections was also indicated in previous studies [12,13,58]. Horses grazing on pasture appeared to have similarly high concentrations of total SCFA and acetate in the dorsal colon as in the ventral colon, which could indicate an ongoing high fibre fermentation in the lower sections of the colon. It is hypothesised that this is attributed to the microbial community being specifically adapted to its environment. Our results confirm the findings of Weinert-Nelson et al. [59], as they also observed higher concentrations of total SCFA, as well as the branched chain SCFA, in faeces of horses grazing on different pastures compared to horses fed hay. As long as no pH drop occurs, a higher production of SCFA in the hindgut is considered as a health benefit.

The type of organic acids produced by microbial fermentation plays a certain role in maintaining equine gastric and intestinal health. Nadeau et al. [10] observed in an *in vitro* study that, in the gastric squamous mucosa, organic acids are predominantly undissociated at pH values below 4.8, facilitating their penetration into the epithelium. Increased intercellular fatty acids may reduce intracellular pH drops, impair sodium transport and cell volume regulation, and contribute to swelling and necrosis of the epithelial cells [10]. This effects appears to depend on the chain length of fatty acids, as iso- or n-butyric acid and iso- or n-valeric acid are more likely to pose a risk than acetic and propionic acids [10]. This is also confirmed by Cehak et al. [60], who reported pH-independent detrimental effects of elevated n-butyrate concentrations on gastric mucosa *in vitro*. In the present study, gastric digesta of horses in the OS2 group contained higher concentrations of n-valerate, whereas horses in the OS1 group showed increased iso-SCFA in the gastric digesta. These SCFA are indirectly linked to proteolytic activity of the microbiota [61,62]. Therefore, their increase could be attributed to the precaecal digestible crude protein (pcdCP) content of the oats used, which contained 92 g pcdCP/kg DM, compared to 49 and 39 g pcdCP/kg DM in hay and matured pasture, respectively [26].

Horses in the PST group exhibited higher gastric n-butyrate concentrations than those in the other feeding groups. Although elevated n-butyrate concentrations impaired the integrity of the glandular gastric mucosa in an *in vitro* study by Cehak et al. [60], no histopathological alterations were observed in the nonglandular mucosa. The n-butyrate concentrations measured in gastric contents in the present study were generally lower than those applied in the Ussing chamber experiment in PNG (10–32 mmol/L) but partially overlapped with the lower concentration range tested in PG (2.5 to 10 mmol/L). In other animals, mainly rodents, n-butyrate has been linked to gastroprotective effects, such as reduced inflammation and improved mucosal repair [63–65]. However, the Ussing chamber model primarily reflects direct effects of n-butyrate on epithelial barrier function and does not account for complex *in vivo* responses, such as modulation of inflammatory mediators, oxidative stress pathways and mucosal repair processes that may contribute to these effects.

As mentioned, SCFA and especially n-butyrate play a fundamental role in maintaining intestinal health. The main functions include provision of energy for colonocytes and promotion of the integrity of the intestinal barrier by stimulating epithelial proliferation, the formation of tight junctions and mucus production [2,3,48,66]. Butyrate production seems to increase when readily fermentable fibre and nonstructural carbohydrates are balanced [3,67,68]. In this context, it seems to be advantageous that in this study, the horses kept on pasture had a higher concentration of n-butyrate in the digesta, especially in parts of the colon.

In the hindgut, oat supplementation at the applied dosages had no apparent effect on the microbial metabolite production, as no significant differences were observed between hay rations with or without oats. This may have been driven by a high precaecal digestibility of oat starch, which can approach up to nearly 100% [20,69,70] and remains high even at larger intake levels [71,72]. However, shifts in microbial composition do not necessarily result in altered concentrations of microbial metabolites. This can be attributed to functional redundancy in the microbiome, whereby taxonomically distinct different microbial communities can exhibit similar metabolic functions and metabolite profiles [73].

The significant differences observed in the hindgut were related to the grazing horses. They showed higher concentrations of n-valerate, iso-SCFA and ammonia in digesta, particularly in sections caudal to the pelvic flexure. The concentrations of these parameters in digesta in the HAY, OS1 and OS2 groups were in a similar range as found by Dougal et al. [54], where mixed rations were fed. Elevated concentrations of these metabolites are considered consistent with increased amino acid and peptide fermentation, in particular with leucine, isoleucine, proline and valine [61,62,74,75]. The metabolised amino acids can be either endogenous or microbial in origin, or may result from feed proteins that have passed through the small intestine undigested. The concentrations of the metabolites are affected by production, microbial utilization and absorption but also by the availability of fermentable carbohydrates and the pH value [76,77]. Moreover, ammonia in digesta is additionally influenced by blood-derived urea recycling, which enters the intestinal lumen and is converted into ammonia by bacterial ureases [77,78]. Despite the comparatively low crude protein concentrations in the sample taken from pasture close to euthanasia (76 g/kg DM) [26], it can be expected that the horses still consumed considerable amounts of protein. Especially on overgrown pastures, as observed at the end of the feeding period, horses are very selective in their feed intake and generally prefer fresh grown, protein- and energy-rich plants [79]. As posited by Edouard et al. [80], the digestible protein concentration of plants has been shown to be the most accurate predictor of which plants are preferred by horses. As horses of the PST group showed higher crude protein concentrations in digesta along the GIT (unpublished data), a higher protein intake on pasture may have contributed to the observed metabolite profile. In ruminants, iso-SCFA stimulate the proliferation of cellulolytic bacteria and anaerobic fungi and improve cellulolytic enzyme activity and fibre degradability [81–83]. Assuming this is similar in horses, it explains the observation that horses grazing on pasture exhibited higher concentrations of total SCFA, with at least numerically higher acetate concentrations in the lower colon. Nevertheless, the elevated ammonia concentrations represent an additional metabolic burden, as ammonia that is absorbed from the GIT needs to be detoxified in the liver.

These results, along with the clustering in the PCA (Fig 2A) and the higher (C2 + C4)/C3 ratio (Table 4) in the distal hindgut, suggest that the pelvic flexure divides the hindgut into two regions with distinct differences in the microbial environment and fermentation activities. Similar observations were also reported by other authors, who observed alterations in the (core) bacterial community when comparing the proximal and distal hindgut [55,56]. The pelvic flexure is an anatomical narrowing between the ventral and the dorsal colon and is considered an important anatomical landmark associated with changes in lumen diameter and flow characteristics of the digesta. It is furthermore described as potential site of altered transit and impaction formation [84–86]. Taken together, these anatomical and functional characteristics suggest that the pelvic flexure may represent a transition zone where changes in digesta flow, substrate availability and microbial activity occur. This contributes to the differences in metabolite profiles observed between the proximal and distal sections of the colon. Consequently, the accuracy of faecal samples in reflecting the conditions in the proximal hindgut is questionable.

Only few studies have examined lactate concentrations in the equine stomach. Lactate is an organic acid produced by microbial fermentation of readily fermentable carbohydrates and is therefore used as an indicator for high amounts of these nutrients in the ration. The concentration of lactate in the GIT reflects the balance between lactate-producing bacteria such as some *Lactobacillus* spp*.* and *Streptococcus* spp*.* and lactate-utilising bacteria such as species of the genera *Veillonella* and *Megasphaera* [87–89]. In our results, the lactate concentration in the foregut tended to be increased on pasture. By the end of the feeding period, the pasture had become overgrown. However, it is assumed that the horses on pasture have selectively consumed mainly young plants with potentially high fructan concentrations, resulting in elevated lactate levels. An increase of lactate with concentrates fed, as documented in other studies [16,44], did not occur. The higher substrate availability for lactate-producing bacteria in the foregut compared to the hindgut and lactate-utilising bacteria converting lactate into SCFA (mainly propionate), explains the barely measurable concentrations in the hindgut [21,35]. The lactate concentrations in the foregut in our study, ranging from an average of 3.9 mmol/L in JEJ to 57.1 mmol/L in PNG are similar to those reported by Glatter et al. [35] and Argenzio et al. [90], but much higher than the results of De Fombelle et al. [15] and Varloud et al. [91], which ranged from 0.3 to 8.0 mmol/L.

Regardless of the parameter, we assume that measurements in the precaecal sections reflect the impact of the morning meals, including the oat meals in OS1 and OS2. In contrast, changes observed in the hindgut sections are more likely related to the diet consumed during the previous day, as described by De Fombelle et al. [15]. This could explain the negligible effect of oat supplementation on the hindgut observed in this study, where the *ad libitum* access to hay may have a protective effect. Thus, further investigations are necessary to detect potential alterations in the microbial composition, because such are not necessarily reflected in the microbial metabolites due to functional redundancy.

Nevertheless, the limitations of the present study should be considered. The relatively small sample size may have reduced the statistical power to detect subtle differences between the groups. Furthermore, the post mortem sampling represents a potential limitation. Although digesta sampling was performed immediately after euthanasia, early post mortem alterations in microbial metabolite concentrations and pH values cannot be completely excluded [92,93]. However, all horses were sampled under comparable conditions and within one hour after euthanasia, thereby reducing the potential influence for post mortem-related alterations and potential systematic bias. Although significant period effects were observed for selected parameters, period was included as a fixed effect in the statistical model. This allowed the effects of ration and gastrointestinal sections to be estimated while accounting for potential environmental variations.

## Conclusion

The results of this experiment demonstrate that the inclusion of oats into the ration alters the microbial metabolite profile in the equine foregut, even when horses have *ad libitum* access to forage. Oat supplementation mainly affected concentrations of n-valerate, iso-SCFA and ammonia in the stomach, suggesting changes in protein fermentation processes. Horses grazing on pasture exhibited metabolic adaptations consistent with enhanced microbial fermentation, including increased production of total SCFA, specifically n-butyrate. In addition, the metabolite profile suggests altered processes associated with microbial protein metabolism in the hindgut of pasture-grazing horses, particularly in the sections caudal to the pelvic flexure.

## Supporting information

S1 Table Raw data of dry matter content (g/kg), pH value, ammonia (mmol/L), individual SCFA (mmol/L) and lactate (mmol/L). (XLSX)

S2 Table Molar percentages of individual SCFA in digesta along the gastrointestinal tract of horses allocated to different rations. (DOCX)

S3 Table *P*-values of F-test displaying the effect of ration and gut section on the molar percentages of individual SCFA. (DOCX)
