# Supplementary material for: Characterisation of microbial metabolite profiles across the gastrointestinal tract of horses fed on pasture, hay or oat-supplemented rations
Source: PLoS One. 2026 Sep 28;21(9):e0359277. doi: 10.1371/journal.pone.0359277 (PMC13619006; doi:10.1371/journal.pone.0359277)
Supplement: Supplementary file 1 [file pone.0359277.s002.docx]

**S2 Table. Molar percentages of individual SCFA in digesta along the gastrointestinal tract of horses allocated to different rations.**

|  |  | **PST** | **HAY** | **OS1** | **OS2** |
| --- | --- | --- | --- | --- | --- |
| acetate | PNG | **84.9** (9.32) | **96.5** (1.97) | **95.6** (3.03) | **94.7** (1.88) |
|  | PG | **81.0** (11.2) | **94.1** (3.18) | **96.0** (1.48) | **93.0** (5.34) |
|  | JEJ | **92.6** (0.76) | **90.8** (2.80) | **96.5** (1.97) | **95.7** (3.45) |
|  | CAE | **68.3** (1.25) | **72.1** (1.88) | **70.4** (1.13) | **70.8** (0.82) |
|  | CVD | **67.7** (0.80) | **69.9** (2.63) | **69.5** (2.66) | **69.8** (1.72) |
|  | CVS | **66.4** (0.79) | **70.3** (2.82) | **69.3** (1.02) | **70.6** (3.68) |
|  | PFL | **68.0** (2.46) | **72.4** (3.74) | **71.1** (2.20) | **71.5** (3.15) |
|  | CDS | **72.2** (1.22) | **75.4** (3.44) | **75.6** (2.48) | **73.4** (3.71) |
|  | CDD | **71.4** (1.39) | **75.2** (3.13) | **76.2** (2.61) | **73.5** (4.45) |
|  | CTR | **71.0** (1.52) | **75.7** (2.94) | **74.9** (1.45) | **74.9** (4.59) |
|  | REC | **73.4** (3.01) | **77.3** (2.86) | **75.5** (3.03) | **75.0** (3.87) |
| propionate | PNG | **7.4** (7.2) | **2.1** (1.5) | **2.2** (2.1) | **2.8** (1.4) |
|  | PG | **2.3** (1.7) | **3.3** (2.3) | **1.4** (0.8) | **3.4** (3.6) |
|  | JEJ | **n.d.** | **n.d.** | **n.d.** | **n.d.** |
|  | CAE | **22.2** (0.77) | **19.7** (1.79) | **20.9** (1.32) | **20.5** (0.91) |
|  | CVD | **19.7** (2.21) | **18.3** (1.97) | **18.8** (2.77) | **18.4** (1.71) |
|  | CVS | **20.0** (2.12) | **18.0** (2.88) | **19.5** (0.34) | **17.8** (4.93) |
|  | PFL | **18.6** (3.78) | **17.5** (3.49) | **18.9** (2.13) | **18.1** (4.58) |
|  | CDS | **14.9** (1.47) | **14.8** (2.96) | **14.7** (2.39) | **16.6** (3.32) |
|  | CDD | **15.9** (1.43) | **15.0** (2.72) | **14.0** (2.59) | **16.4** (3.24) |
|  | CTR | **16.7** (0.63) | **16.1** (2.86) | **17.1** (1.72) | **16.7** (4.66) |
|  | REC | **16.7** (2.88) | **15.3** (2.62) | **17.0** (3.08) | **16.1** (4.57) |
| n-butyrate | PNG | **7.6^a^** (4.5) | **0.9^b^** (0.8) | **1.0^b^** (1.1) | **1.2^b^** (0.86) |
|  | PG | **16.6^a^** (9.8) | **1.9^b^** (1.4) | **0.8^b^** (0.9) | **2.4^b^** (2.2) |
|  | JEJ | **7.4^a^** (0.76) | **9.2^a^** (2.8) | **3.5^b^** (2.0) | **4.3^ab^** (3.5) |
|  | CAE | **8.4** (1.3) | **7.2** (0.59) | **7.5** (0.52) | **7.5** (1.0) |
|  | CVD | **10.3** (0.78) | **9.4** (1.9) | **9.0** (2.2) | **9.1** (1.3) |
|  | CVS | **10.4** (1.4) | **8.9** (2.4) | **8.4** (0.94) | **7.4** (0.49) |
|  | PFL | **8.3** (0.38) | **6.5** (1.5) | **6.9** (0.40) | **6.6** (0.72) |
|  | CDS | **6.4** (1.2) | **5.4** (1.1) | **5.6** (0.73) | **6.4** (2.2) |
|  | CDD | **6.5** (1.4) | **5.4** (0.78) | **5.6** (0.34) | **6.4** (2.2) |
|  | CTR | **6.5** (1.5) | **4.9** (0.62) | **4.8** (0.61) | **5.2** (0.54) |
|  | REC | **5.1^ab^** (0.94) | **4.2^b^** (0.36) | **4.8**^ab^ (0.51) | **5.3^a^** (0.44) |
| n-valerate | PNG | **0.0^c^** (0.0) | **0.1^bc^** (0.1) | **0.1^ab^** (0.1) | **0.2^a^** (0.2) |
|  | PG | **0.0^b^** (0.0) | **0.1^a^** (0.1) | **0.0^b^** (0.0) | **0.1^ab^** (0.1) |
|  | JEJ | **n.d.** | **n.d.** | **n.d.** | **n.d.** |
|  | CAE | **0.4** (0.0) | **0.4** (0.1) | **0.4** (0.1) | **0.4** (0.1) |
|  | CVD | **0.6** (0.2) | **0.6** (0.1) | **0.5** (0.1) | **0.6** (0.1) |
|  | CVS | **0.8^ab^** (0.2) | **0.6^b^** (0.1) | **0.6^ab^** (0.1) | **0.8^a^** (0.2) |
|  | PFL | **1.0^a^** (0.24) | **0.7^b^** (0.2) | **0.6^b^** (0.1) | **0.7^b^** (0.2) |
|  | CDS | **1.3^a^** (0.29) | **0.8^b^** (0.2) | **0.7^b^** (0.1) | **0.6^b^** (0.04) |
|  | CDD | **1.2^a^** (0.28) | **0.7^b^** (0.2) | **0.7^b^** (0.1) | **0.7^b^** (0.1) |
|  | CTR | **1.1^a^** (0.12) | **0.5^b^** (0.1) | **0.5^b^** (0.1) | **0.6^b^** (0.1) |
|  | REC | **0.8^a^** (0.1) | **0.5^b^** (0.1) | **0.5^b^** (0.1) | **0.5^ab^** (0.3) |
| iso-SCFA | PNG | **0.1^b^** (0.1) | **0.5^ab^** (0.2) | **1.1^a^** (1.4) | **1.1^a^** (0.80) |
|  | PG | **0.1^c^** (0.1) | **0.5^b^** (0.2) | **1.8^a^** (0.83) | **1.2^a^** (0.63) |
|  | JEJ | **n.d.** | **n.d.** | **n.d.** | **n.d.** |
|  | CAE | **0.7** (0.1) | **0.6** (0.2) | **0.8** (0.1) | **0.8** (0.2) |
|  | CVD | **1.7** (1.11) | **1.9** (0.30) | **2.1** (0.29) | **2.2** (0.78) |
|  | CVS | **2.4^ab^** (0.94) | **2.1^b^** (0.21) | **2.3^a^** (0.25) | **3.3^ab^** (1.43) |
|  | PFL | **4.0^a^** (1.33) | **2.9^ab^** (0.55) | **2.5^b^** (0.28) | **3.2^ab^** (0.95) |
|  | CDS | **5.3^a^** (0.62) | **3.6^b^** (0.45) | **3.3^bc^** (0.45) | **3.0^c^** (0.61) |
|  | CDD | **5.1^a^** (0.47) | **3.7^b^** (0.64) | **3.4^bc^** (0.44) | **3.0^c^** (0.68) |
|  | CTR | **4.7^a^** (0.13) | **2.8^b^** (0.34) | **2.6^b^** (0.46) | **2.7^b^** (0.47) |
|  | REC | **4.1^a^** (0.78) | **2.7^b^** (0.74) | **2.3^b^** (0.33) | **3.0^ab^** (0.64) |

Rations: PST, pasture 24 h/d (n = 4); HAY, hay *ad libitum* (n = 8); OS1, hay *ad libitum* and oats at 1 g starch/kg BW/meal (n = 6); OS2, hay *ad libitum* and oats at 2 g starch/kg BW/meal (n = 5). Sections: PNG, gastric pars nonglandularis; PG, gastric pars glandularis; JEJ, jejunum; CAE, caecum; CVD, colon ventrale dextrum; CVS, colon ventrale sinistrum; PFL, felxura pelvina; CDS, colon dorsale sinistrum; CDD, colon dorsale dextrum; CTR, colon transversum; REC, rectum. Abbreviations: BW, body weight; n.d., not detectable; SCFA, short chain fatty acids. ^abc^Superscripts indicate differences among the rations within section with P < 0.05. Standard deviation is given in brackets.
