# Supplementary material for: Characterisation of microbial metabolite profiles across the gastrointestinal tract of horses fed on pasture, hay or oat-supplemented rations
Source: PLoS One. 2026 Sep 28;21(9):e0359277. doi: 10.1371/journal.pone.0359277 (PMC13619006; doi:10.1371/journal.pone.0359277)
Supplement: Supplementary file 2 [file pone.0359277.s003.docx]

**S3 Table. *P*-values of F-test displaying the effect of ration and gastrointestinal section on the molar percentages of individual SCFA.**

|  | ration | section | ration*section |
| --- | --- | --- | --- |
| acetate | 0.289 | < 0.001 | 0.100 |
| propionate | 0.573 | < 0.001 | 0.386 |
| n-butyrate | < 0.001 | < 0.001 | 0.001 |
| n-valerate | 0.076 | < 0.001 | < 0.001 |
| iso-SCFA | 0.182 | < 0.001 | < 0.001 |

Abbreviations: SCFA, short chain fatty acids.
